# SERIneALanine Killer: SPT promiscuity inhibits tumour growth via intra-tumoral deoxysphingolipid production

**DOI:** 10.1038/s41392-020-00401-6

**Published:** 2020-11-24

**Authors:** Mattia Falcone, Oliver D. K. Maddocks

**Affiliations:** University of Glasgow Institute of Cancer Sciences, Wolfson Wohl Cancer Research Centre, Garscube Estate, Glasgow, G61 1QH UK

**Keywords:** Cancer metabolism, Cancer therapy

In a recent article in Nature, Muthusami et al.^1^ highlight the promiscuity of serine palmitoyltransferase (SPT) for non-essential amino acids under low serine conditions, illustrating a previously unappreciated mechanism for inhibition of tumour growth—increased levels of deoxysphingolipids.

Tumour cells acquire a range of metabolic adaptations, driven directly and indirectly by genetic mutations, making altered cellular metabolism a hallmark of cancer.^2^ Metabolic rewiring drives tumour progression by allowing cancer cells to take advantage of and shape their niche in order to command a constant nutrient supply. However, cancer-specific metabolic adaptations also introduce vulnerabilities that can be exploited clinically. Since the implementation over a half-century ago of chemotherapeutic anti-metabolite drugs such as methotrexate, a folate cycle inhibitor, new pharmacological approaches focussed on targeting metabolic pathways are in trial, and, recently, novel defined dietary regimens aimed to aid cancer therapy have come to the fore.^3^ In particular, modulation of serine and glycine availability is proven to be effective in inhibiting the growth of specific subtypes of tumour in vivo.^4^ These non-essential amino acids, which can be synthesised from glucose, are pivotal substrates for several metabolic pathways, including the biosynthesis of proteins and nucleic acids. However, the molecular mechanism behind the ability of serine-free diet to restrict tumour growth, with respect to alteration in the cancer lipodome remains uncharted. In a recent publication in *Nature*, Muthusami et al.^1^ explore the effect of tumour serine restriction on sphingolipid heterogeneity, highlighting the promiscuity of SPT as a metabolic liability of cancer cells that can be exploited through diet or pharmacological means.

The authors began by comparing the metabolic changes induced in cancer cell lines by attachment-independent (spheroid) growth, finding increased secretion and intracellular accumulation of alanine along with a decreased flux through the pyruvate dehydrogenase enzyme. Since previous reports have shown that inhibition of the mitochondrial pyruvate carrier (MPC) is able to decrease alanine levels in adherent cultured cells, to further investigate the correlation between the alanine accumulation and altered mitochondrial metabolism, Muthusami et al.^1^ performed [U-^13^C_6_]glucose and [2,3-^13^C_2_]alanine tracing in both adherent and spheroid cultures. Interestingly, upon MPC inhibition or knockdown, the authors observed that spheroid growth was boosted, secretion/accumulation of alanine was rescued and serine synthesis increased. Specifically, the authors demonstrated in spheroid cultures that while glucose metabolism was diverted towards the serine synthesis pathway, alanine flux into the TCA cycle was increased, bypassing the inactivity of MPC. Hence, Muthusami et al.^1^ hypothesised that alanine could be deleterious for anchorage-independent growth; the authors showed that the advantage given by the lack of the MPC was abrogated by exogenous alanine supplementation. Likewise, a comparable effect was achieved by serine/glycine depletion, suggesting a connection between the two events.

**Fig. 1:**
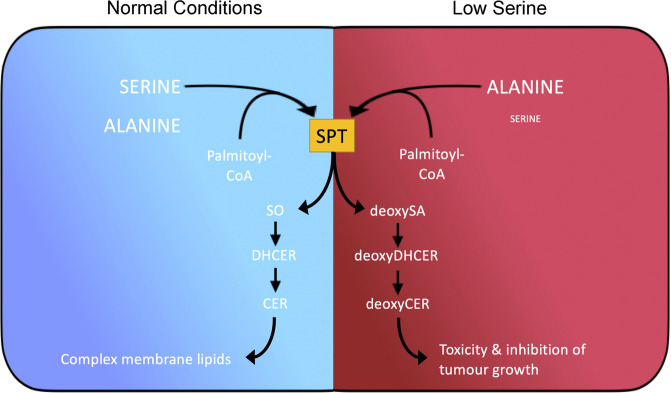
SPT promiscuity links intra-tumoral serine and alanine metabolism to sphingolipid heterogeneity in cancer cells. Schematic diagram depicting sphingolipid synthesis under normal (left, blue) and low serine (red, right) conditions. Under normal conditions serine palmitoyltransferase (SPT) synthesises sphingosine (SO) as the canonical precursor of dihydroceramide (DHCER), ceramide (CER) and ultimately complex membrane lipids. Under low serine conditions alanine is an SPT substrate, fuelling synthesis of 1-deoxysphinganine (deoxySA), which is further metabolised into 1-deoxydihydroceramide (deoxyDHCER) and 1-deoxyceramide (deoxyCER), altering membrane lipid diversity and impairing tumour growth

To further elucidate the mechanism by which serine restriction impaired spheroid growth, Muthusami et al.^1^ compared the kinetics of different serine-consuming enzymes and SPT displayed a *K*_*m*_ within intratumoral serine concentrations, thus limiting its affinity for serine in serine-restricted conditions. Under normal conditions SPT catalyses sphingosine synthesis using serine as a substrate. However, SPT displays substrate promiscuity, and during serine restriction can switch to alanine (Fig. 1). Using alanine, SPT generates 1-deoxysphinganine, a precursor of 1-deoxydihydroceramide (deoxyDHCER) and 1-deoxyceramide (deoxyCER). By either exogenous supplementation of those lipids or inhibition of their metabolism at different stages, the authors elegantly demonstrated that the negative growth phenotype observed in the serine-starved spheroids culture was, at least in part, due to toxicity of deoxyCER.

Next, the authors investigated this newly identified vulnerability in the context of in vivo physiology using xenograft models coupled with pharmacological and dietary intervention. In similarity to recent studies, the authors demonstrated that upon serine and glycine starvation tumour progression was significantly decreased.^4^ Mechanistically this was accompanied by an increase in toxic deoxysphingolipids, similar to the in vitro phenotype initially shown, whereas other canonical sphingolipids maintained stable levels. Intriguingly, deoxysphingolipids increased to a lesser extent in healthy tissues, such as the liver, indicating that the tumour cells are more prone to produce and retain the toxic lipid species than normal cells. Additionally, the authors also reported decreased levels of lactosylceramide (d18:1/d16:0), a precursor of complex membrane lipids, suggesting an altered membrane composition. Furthermore, the authors showed increased expression of the liver enzymes involved in alanine degradation, suggesting putative alanine scavenging activity of the liver and prevention of the accumulation of toxic lipid species.

To validate the proposed mechanism, Muthusami et al.^1^ rescued in vivo tumour growth by inhibiting SPT concurrent with a serine and glycine-restricted diet. While at high dose the SPT inhibitor displayed a systemic harmful effect, at lower drug concentration the authors observed stable liver function and restored xenograft growth despite limited serine availability. Finally, the authors employed an alternative means to decrease serine availability, by administering a phosphoglycerate dehydrogenase (PHGDH, first enzyme of the serine synthesis pathway) inhibitor that significantly reduced both plasma and tumour levels of serine and glycine concomitantly with toxic deoxysphingolipid accumulation.

This study has elegantly highlighted the possibility of exploiting promiscuous enzymes such as SPT to treat cancer by limiting the availability of the primary substrate, leading to the production of toxic lipid species, altering cellular membrane composition and ultimately impairing tumour growth. Importantly, it was demonstrated that tumour cells were more susceptible to this intervention than healthy tissues, giving a clear therapeutic window for this approach. Given the importance of several other serine/glycine-dependent pathways in cancer cell metabolism,^5^ such as protein synthesis, folate-dependent one-carbon metabolism (for nucleotide synthesis) and glutathione synthesis, it seems likely that toxic metabolite accumulation is one of several metabolic outcomes of serine and glycine limitation that could contribute concurrently to impeding tumour growth. It is technically challenging to dissect the exact contribution of each process as inhibiting/supplementing one metabolic pathway will likely modulate the serine availability for use by alternate pathways, making it difficult to isolate the importance of any individual pathway. Interestingly, the promiscuity of SPT is not limited to serine and alanine, but also to palmitoyl-CoA and other acyl-CoA species, suggesting a more intricate and complex network of stress signals via tuning cellular lipid diversity that could prompt further research for new therapies.
